# Evaluation of interventions for essential newborn care practices at home-based delivery in India

**DOI:** 10.1093/eurpub/ckac130.038

**Published:** 2022-10-25

**Authors:** J Moon, G Usmanova, J van der Schans, JW Noh, R Biesma

**Affiliations:** 1 Graduate School of Medical Sciences, University of Groningen, Groningen, Netherlands; 2 Monitoring, Evaluation and Research, Jhpiego, New Delhi, India; 3 Department of Health Sciences, University of Groningen, Groningen, Netherlands; 4 Faculty of Economics and Business, University of Groningen, Groningen, Netherlands; 5 Division of Health Administration, Yonsei University, Seoul, South Korea; 6 Department of Health Science, Global Health, University of Groningen, Groningen, Netherlands

## Abstract

India has the largest share of neonatal mortality, accounting for 21.7 per 1,000 in 2019, while the global goal is at least 12 per 1,000 by 2030. More than 20% of deliveries still occur at home in India for various reasons. Several national interventions were designed to ensure essential practices even at home by engaging skilled birth attendants (SBAs), antenatal care (ANC), and community health workers (CHWs). This study evaluates the effectiveness of these interventions on essential newborn care practices (EP); clean cord care, thermal care, and breastfeeding. Using data from the 2015-2016 India Demographic and Health Survey (n = 9,273), this study employs structural equation modeling to confirm the relationship between SBA, ANC, CHWs` counseling, and EP, including indirect effects of ANC and CHWs and moderating effects of women's empowerment. The results show that SBA and ANC have significant direct effects (standardised coefficient=0.105 and 0.056, respectively) on EP, and ANC and CHWs have significant indirect effects (0.015 and 0.004) in the well-fitted model (CFI=0.938, TLI=0.920, RMSEA (upper 90% CI)=0.028 (0.029), SRMR=0.044). The empowerment-related factors which had a significant positive moderating effect on the paths from SBA to EP and from ANC to EP are decision-making power (0.007, 0.003), allowed mobility (0.002, 0.001), and education (0.009, 0.004). More than 90% of EP variance is not associated with the factors in this model (standardised coefficient=0.958). SBA demonstrated the most considerable effectiveness for EP, while ANCs and CHW indirectly impacted EP. Improving women's empowerment can be an effective strategy to enhance EP. Previous literature said that the other factors explaining EP could be quality of care, other interventions for a safe birth, and cultural characteristics. Policymakers are recommended to consider comprehensive factors to address barriers to safe home birth and design CHW's intervention to persuade SBA.

**Key messages:**

- To optimize the effects of skilled birth attendants, quality of care need to be improved.
- Improving women's empowerment can be an effective strategy to enhance essential practices.

